# Group physical therapy for knee osteoarthritis: protocol for a hybrid type III effectiveness-implementation trial

**DOI:** 10.1186/s43058-023-00502-7

**Published:** 2023-10-12

**Authors:** Sara Webb, Connor Drake, Cynthia J. Coffman, Caitlin Sullivan, Nina Sperber, Matthew Tucker, Leah L. Zullig, Jaime M. Hughes, Brystana G. Kaufman, John A. Pura, Livia Anderson, Susan N. Hastings, Courtney H. Van Houtven, Lauren M. Abbate, Helen Hoenig, Lindsay A. Ballengee, Virginia Wang, Kelli D. Allen

**Affiliations:** 1 Center of Innovation to Accelerate Discovery and Practice Transformation, Durham VA Health Care System, Durham, NC USA; 2 Department of Population Health Sciences, Duke University School of Medicine, Durham, NC USA; 3 Department of Biostatistics and Bioinformatics, Duke University School of Medicine, Durham, NC USA; 4 Department of Implementation Science, Wake Forest University School of Medicine, Winston-Salem, NC USA; 5 Section On Gerontology and Geriatric Medicine, Division of Internal Medicine, Wake Forest School of Medicine, Winston-Salem, NC USA; 6 Margolis Center for Health Policy, Duke University, Durham, NC USA; 7 AstraZeneca, Durham, NC USA; 8 Center for the Study of Aging and Human Development, Duke University School of Medicine, Durham, NC USA; 9 Geriatric Research, Education, and Clinical Center, Durham VA Health Care System, Durham, NC USA; 10 Department of Medicine, Duke University School of Medicine, Durham, NC USA; 11 VA Eastern Colorado Geriatric Research Education and Clinical Center and University of Colorado School of Medicine, Aurora, CO USA; 12 Physical Medicine and Rehabilitation Services, Durham VA Health Care System, Durham, NC USA; 13 Department of Medicine & Thurston Arthritis Research Center, University of North Carolina at Chapel Hill School of Medicine, Chapel Hill, NC USA

**Keywords:** Osteoarthritis of knee, Implementation science, Group physical therapy, Physical therapy, Functional independence, Veterans

## Abstract

**Background:**

Knee osteoarthritis (OA) is a leading cause of chronic pain and disability and one of the most common conditions treated in outpatient physical therapy (PT). Because of the high and growing prevalence of knee OA, there is a need for efficient approaches for delivering exercise-based PT to patients with knee OA. A prior randomized controlled trial (RCT) showed that a 6-session Group Physical Therapy Program for Knee OA (Group PT) yields equivalent or greater improvements in pain and functional outcomes compared with traditional individual PT, while requiring fewer clinician hours per patient to deliver. This manuscript describes the protocol for a hybrid type III effectiveness-implementation trial comparing two implementation packages to support delivery of Group PT.

**Methods:**

In this 12-month embedded trial, a minimum of 16 Veterans Affairs Medical Centers (VAMCs) will be randomized to receive one of two implementation support packages for their Group PT programs: a standard, low-touch support based on Replicating Effective Programs (REP) versus enhanced REP (enREP), which adds tailored, high-touch support if sites do not meet Group PT adoption and sustainment benchmarks at 6 and 9 months following launch. Implementation outcomes, including penetration (primary), adoption, and fidelity, will be assessed at 6 and 12 months (primary assessment time point). Additional analyses will include patient-level effectiveness outcomes (pain, function, satisfaction) and staffing and labor costs. A robust qualitative evaluation of site implementation context and experience, as well as site-led adaptations to the Group PT program, will be conducted.

**Discussion:**

To our knowledge, this study is the first to evaluate the impact of tailored, high-touch implementation support on implementation outcomes when compared to standardized, low-touch support for delivering a PT-based intervention. The Group PT program has strong potential to become a standard offering for PT, improving function and pain-related outcomes for patients with knee OA. Results will provide information regarding the effectiveness and value of this implementation approach and a deeper understanding of how healthcare systems can support wide-scale adoption of Group PT.

**Trial registration:**

This study was registered on March 7, 2022 at ClinicalTrials.gov (identifier NCT05282927).

**Supplementary Information:**

The online version contains supplementary material available at 10.1186/s43058-023-00502-7.

Contributions to the field

- This study will evaluate the effectiveness of tailored, high-touch implementation support when compared to standardized, low-touch support for delivering a physical therapy care model in real-world clinical settings.
- This study will evaluate the effects of implementing group physical therapy for knee OA, a model that can improve access and efficiency of care delivery.
- Results will provide a deeper understanding of site-level characteristics and pre-conditions associated with a need for more intensive implementation support.

## Background

Knee OA affects approximately 14 million people in the US [1], and the prevalence is rising [2]. Knee OA is a leading cause of chronic pain and disability [3], and it has negative impacts on many other outcomes including depressive symptoms, sleep problems, work loss, risk for cardiovascular disease, and chronic opioid use [3–8]. Veterans are at substantially greater risk for knee OA, due in part to high rates of joint injuries and activities that place excessive stress on joints [9, 10]. Within the Department of Veterans Affairs (VA) Healthcare System, arthritis is one of the most prevalent health conditions, with knee OA being the most common type [9, 11, 12].

Exercise-based physical therapy (PT) is a key component of guideline concordant knee OA management [13–16]. However, PT is underutilized for knee OA [17, 18], resulting in missed opportunities to improve outcomes for the many patients with this health condition [19–24]. A key challenge is the high demand for PT services for knee OA and limited availability of PT services within some health care settings [25]. This signals a need to develop, test, and implement efficient care models for delivering PT services for knee OA. To address this need, we conducted a RCT comparing group-based PT (Group PT) with traditional, individual PT among Veterans with knee OA [26, 27]. Group PT resulted in equivalent or greater improvements in pain and functional outcomes compared with individual PT [26]. This is important because Group PT requires fewer clinician hours per patient to deliver and therefore has the potential to improve operational efficiency and generate healthcare savings while maintaining comparable patient-level outcomes. Following the RCT, the Durham Veterans Affairs Healthcare System (DVAHCS) offered Group PT as a clinical service, and we found that patients achieved clinically relevant improvements in pain and functional outcomes that were comparable to those observed in the RCT [28].

Despite this evidence base suggesting that Group PT could improve efficiency without compromising effectiveness, there are obstacles to implementing new care models. For example, as observed in the Group PT implementation at the DVAHCS, ongoing communication and education is required across service lines when introducing a new program. In addition, providers need to be aware of the patient eligibility criteria for the program and process for submitting referrals for appropriate patients. For clinicians delivering the program, there may be challenges in learning how to deliver content in a new format (e.g., within a group setting). Therefore, scalable and efficient strategies are required to support sites in adopting and implementing new programs such as Group PT. This paper describes the protocol for a hybrid type III effectiveness-implementation trial that extends our work in studying Group PT by comparing outcomes for two different implementation support packages to promote adoption of this evidence-based program (EBP). This study is part of a larger research project being conducted at the DVAHCS, Function and Independence Quality Enhancement Research Initiative (Function QUERI); funding ID QUE:20–023.

## Methods/design

### Overview

The overall goal of this trial is to evaluate implementation of Group PT using activities informed by Replicating Effective Programs (REP), which has been described as both an implementation framework and strategy [29, 30]. REP was selected because of its efficiency, scalability, and flexibility to facilitate site-specific adaptations for best fit at each facility and patient population. The study involves an embedded parallel cluster RCT in which VA sites are randomized to receive one of two implementation support packages to deliver Group PT: foundational REP (“low-touch”) implementation support only (active comparator) versus enREP (experimental), which adds “high-touch” implementation support for sites that do not meet a priori benchmarks. We hypothesize that sites randomized to receive high-touch support will have superior implementation outcomes, including higher penetration, adoption, and fidelity, at 12 months. An explanatory convergent mixed method design [31] will be used to integrate qualitative data to better understand site implementation context and experience. We will also collect patient-level effectiveness outcomes to examine improvement in patient outcomes (overall and by study arm) and staffing and labor costs to conduct a business case analysis (BCA). The Standards for Reporting Implementation Studies (StaRI) checklist is available as supplemental material [32].

### Randomization

The study will enroll a minimum of 16 VAMC sites. Because site complexity and rurality are hypothesized to affect program implementation outcomes, randomization of sites will be stratified based on these two factors. Site complexity will be determined by a VA facility-level measure that considers the complexity of services provided, with level 1a being the most complex and level 3 being the least complex; we will dichotomize complexity as high: 1a, 1b, 1c versus low: 2, 3, and small facilities/outpatient clinics with no complexity level assigned [33]. Rurality will be based on the VA Office of Rural Health rurality calculator, which uses the closest facility or county/zip code level data to determine percent of rural Veterans served; we will dichotomize rurality as high: sites serving ≥ 50% rural/highly rural Veterans versus low: sites serving < 50% rural/highly rural Veterans. We aim to enroll at least 4 rural sites. Stratified block randomization will be used, with sites randomized 1:1 to REP or enREP; all study team members will be blinded to block size except the statisticians. Randomization results will be revealed to study members 2 weeks prior to the 6-month adoption benchmark assessment. Sites are only notified of their randomization arm if they fail to meet adoption or sustainment benchmarks.

### Study design

**Fig. 1:**
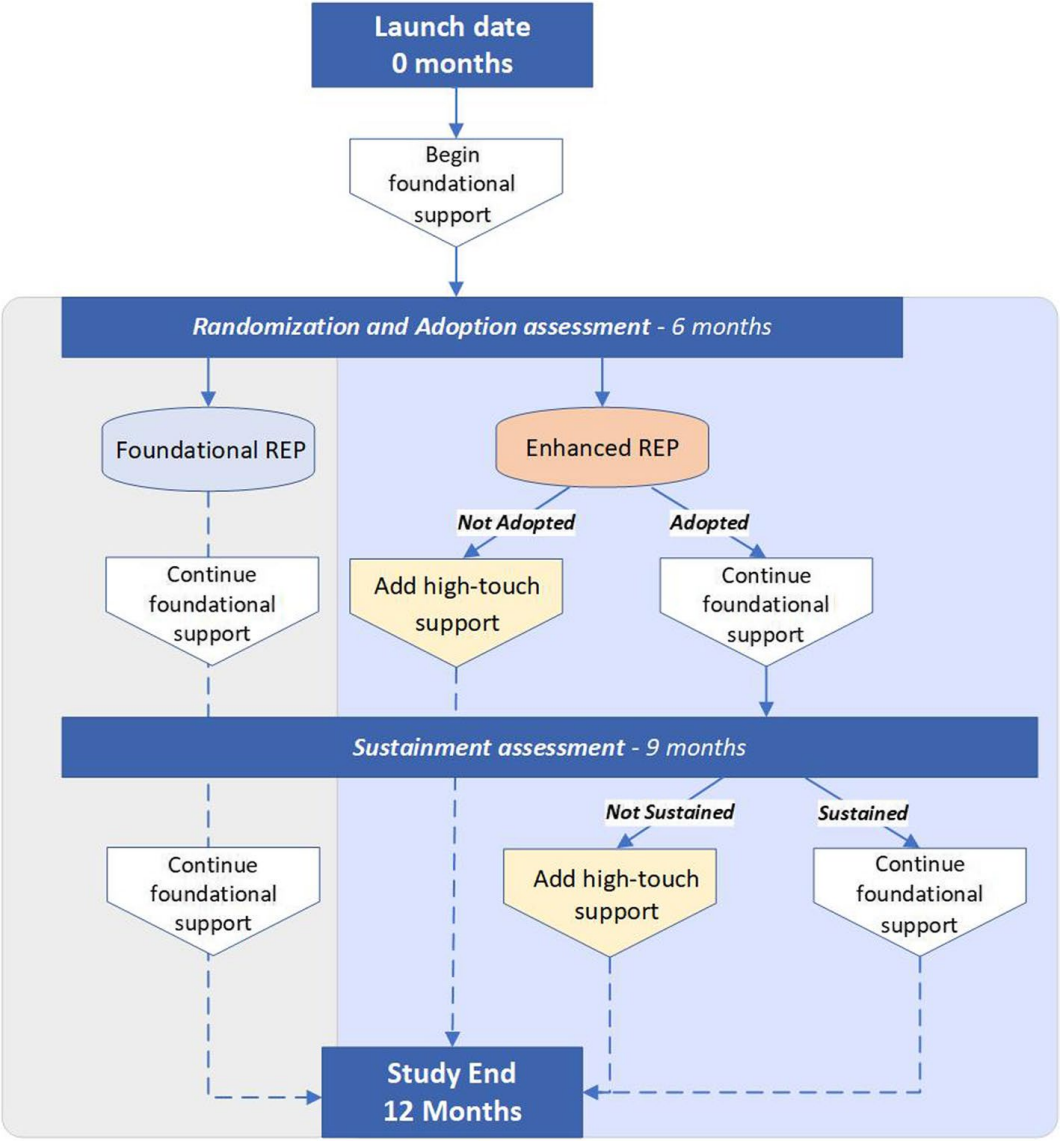
Implementation strategy randomization flowchart

As illustrated in Fig. 1, all sites will receive a minimum “dose” of foundational support for 12 months, beginning immediately after a launch date, which serves as the beginning of the 12-month implementation period. Sites will be evaluated 6 months after launch to assess whether they meet the adoption benchmark of delivering at least one Group PT class and enrolling at least five patients (who must attend at least one Group PT class within the 6-month period). Only sites randomized to the enREP arm who do not achieve the adoption benchmark will be notified of their randomization assignment and start receiving high-touch implementation support. Otherwise, sites will continue with foundational, low-touch support only. A second benchmark assessment will occur 9 months after the launch date to evaluate program sustainment, defined as enrolling 15 new patients between months 7 and 9. Sites randomized to the enREP arm that met the 6-month adoption benchmark but do not meet the sustainment benchmark at 9 months will be notified and begin receiving high-touch support; therefore, sites in the enREP arm may receive 3 or 6 months of high-touch support.

This study was reviewed and approved by the Institutional Review Board of the DVAHCS (#2334). Additionally, all quantitative surveys and qualitative interviews were reviewed by the VA Office of Labor Management Relations, which notified applicable VA national unions.

## Group PT evidence-based program

### Patient eligibility and enrollment

Patients will be eligible for Group PT if they have a clinician diagnosis of symptomatic knee OA and ineligible if they have a substantial fall risk or co-occurring health conditions that would make participation in a group exercise class unsafe. Patients can be referred to the Group PT program by clinicians or self-referred. Once patients are referred to Group PT, a member of the clinical delivery team will conduct an initial evaluation (in person or remotely) to determine patient appropriateness for the program and a starting point for the exercise program.

### Class structure and content

**Fig. 2:**
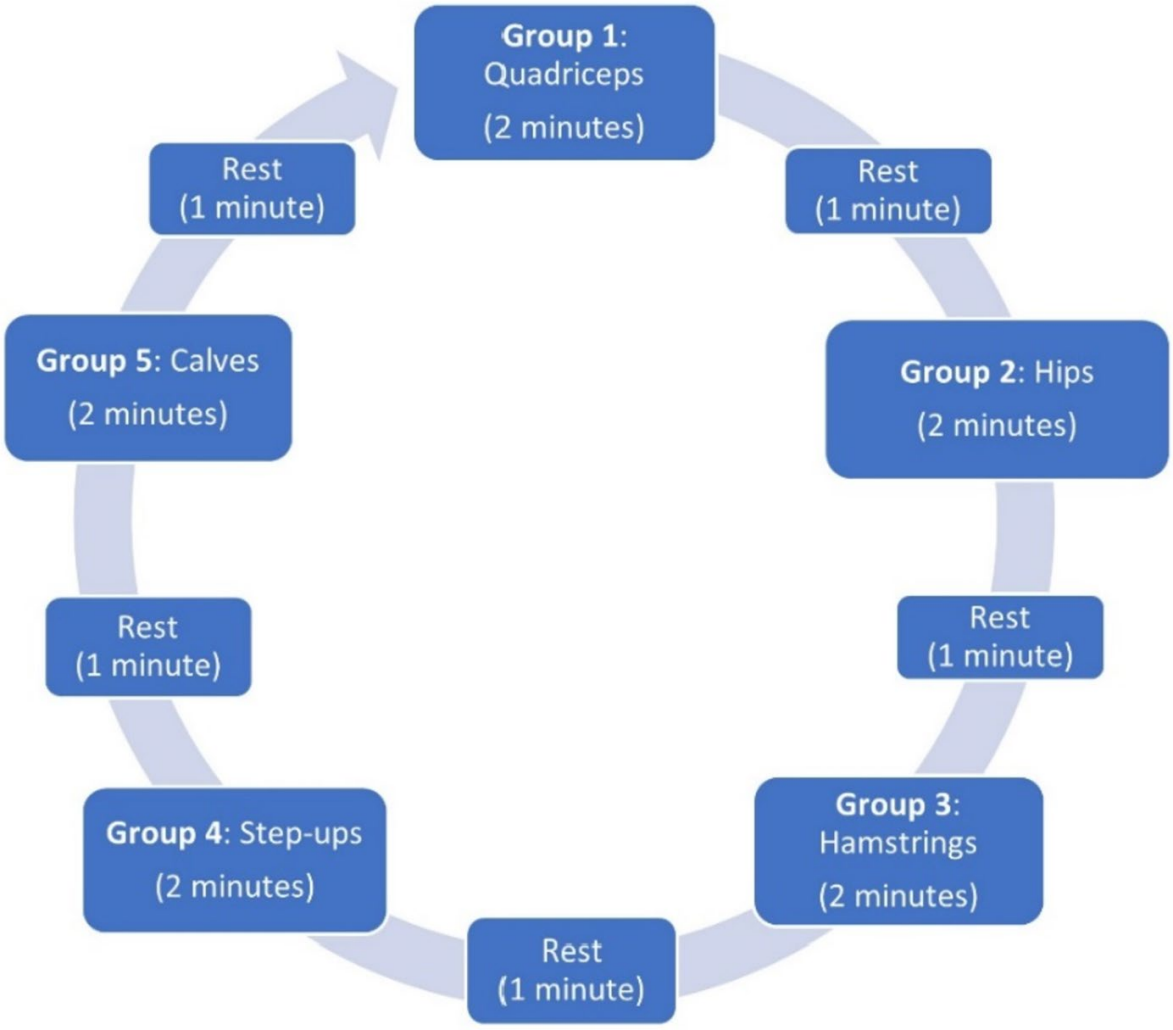
Group PT exercise intervals

Group PT was developed based on our prior work [28], best practices regarding PT and exercise for knee OA [34], and guidance from clinical partners including practicing physical therapists and VA Physical Medicine and Rehabilitation Service (PM&RS) leaders with expertise in PT delivery and telerehabilitation. Sites implementing Group PT must incorporate three essential elements into program delivery: (1) six 1-h sessions with a trained clinician, including strengthening exercises and educational content, (2) offered in a group format, and (3) targeting patients with knee OA. Beyond these essential elements, sites will have flexibility in many aspects of Group PT implementation to fit their needs, including class frequency, number of patients per class (recommendation of ≤ 10), delivery mode (in-person, telehealth, or hybrid), and number of classes offered simultaneously. Each Group PT session will be approximately 1 h beginning with collecting patient outcomes, followed by warm-up and sharing “success stories”, then strengthening exercises, and ending with stretching and OA education. Strengthening exercises are organized into 5 groups, each with “challenge levels” (Table 1) and performed in five intervals, each consisting of 2 min of exercise and 1 min of rest (Fig. 2); two full rotations will be conducted per session. Group PT leaders have flexibility to adjust exercises for individual patient needs. Patients will also be instructed to perform exercises at least two times per week at home. Patients will receive a handbook with exercise guidance and educational modules as well as access to online videos to guide them through each of the exercises.

**Table 1:** Group PT exercises, challenge levels, and suggested activities for warm-up and cool-down

	**LEVEL 1**	**LEVEL 2**	**LEVEL 3**	**LEVEL 4**	**LEVEL 5**
**Group 1** ** (QUADRICEPS)**	Knee extension	Mini-squat	Sit to stand	Weighted squat	Lunge
**Group 2** **(HIPS)**	Seated hip abduction	Standing hip abduction	Standing hip abduction *(with weight or exercise band)*	Crab walk	Crab walk *(with weight or exercise band)*
**Group 3** **(HAMSTRINGS)**	Hamstring curl	Standing hip extension	Deadlift	Dumbbell swing	Single leg deadlift
**Group 4** **(STEP UPS)**	2” step up	4” step up	6” step up	8” step up	10” step up
**Group 5** **(CALVES)**	Bilateral calf raise	Bilateral step calf raise	Single leg calf raise	Single leg calf raise	Single leg step calf raise
**Warm-up** **(NO LEVELS)**	• Walking, Marching in Place • Side-to-Side lunges • Arm circles • Torso rotation				
**Cool-down** **(NO LEVELS)**	• Hamstring (seated, standing) • Quad (standing) • Calf (standing) • Hip Flexor (standing, kneeling) • Lower back (seated) • Thoracic extension (seated) • Thoracic rotation (seated)				

**Recommendation for progression**: patients progress to the next level if they can perform three sets of 15 repetitions and their rating of perceived exertion is < 5 (“hard”) on a scale of 0–10

## Implementation framework and strategies

### Overview

**Fig. 3:**
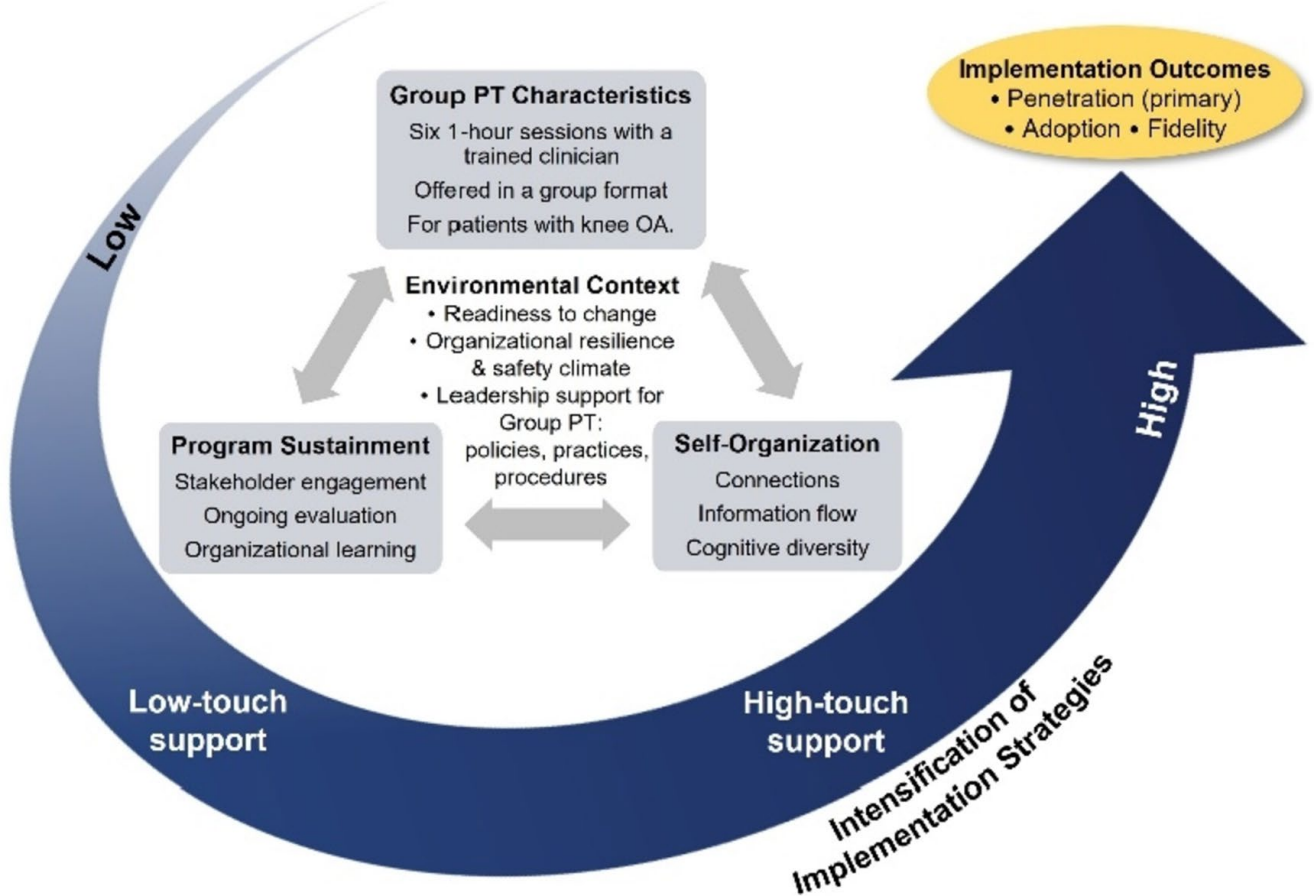
Function QUERI implementation intensification framework

Drawing from existing implementation science and organizational theory frameworks, we designed implementation support materials and activities to promote penetration, adoption, and fidelity of Group PT. Tailoring concepts from the QUERI Implementation Roadmap [35], Dynamic Sustainability Framework [36], complexity science principles, and Proctor’s taxonomy of implementation outcomes [37], our conceptual model (Fig. 3) adapted from Decosimo et al. [38] posits that implementation success is a function of interactions among Group PT intervention characteristics, contextual factors, and team function. To explain this phenomenon, complexity science postulates that the implementing team’s capacity to self-organize and communicate enables the type of problem solving and coordination required to incorporate a novel program into routine practice [39]. Consistent with these theoretical underpinnings, intervention characteristics, system-level implementation determinants (e.g., policies, regulations, and the population being served), team function, and other facets of the practice setting influence adoption and sustainment, but also vary across practice settings, and thus necessitates tailoring implementation to local context. All sites will receive foundational, low-touch implementation support to promote adaptation of Group PT to fit the clinical context in which it is being introduced. We anticipate that this will be sufficient for some but not all sites implementing Group PT. We posit that for some non-adopting sites, high-touch implementation support will be required to effectively facilitate self-organization and problem-solving to achieve improvements in implementation outcomes of interest. For an overview of activities specific to REP and enREP see Table 2.

**Table 2:** Components of low-touch and high-touch support

Package	Activity	Description
Low-touch/foundational REP (resources available to all sites for full study period)	Toolkit	Standardized program materials and training curriculum to educate delivery teams about the Group PT intervention and implementation process, including recorded webinars, implementation handbook, and Group PT delivery guide.
	SharePoint	Secure SharePoint for access to Group PT implementation support materials (e.g., patient materials, marketing templates, guides for documenting patient outcomes), and standardized materials to facilitate monitoring sites’ progress.
	Pre-developed electronic health record (EHR) templates	Consult, initial evaluation, and class participation EHR templates developed with clinical guidance to facilitate Group PT delivery and collection of patient-reported outcomes.
	Data reports	Monthly reports to assist sites with tracking their implementation activity (e.g., patient enrollment, attendance, and satisfaction). Quarterly reports will be sent with patient outcomes (e.g., PROMIS scores and chair rise improvement).
	Learning collaborative	Office hour calls and Microsoft Teams channel designed to capture and share local knowledge through networking. Office hour calls will be specific to each cohort of sites; they will include a short, structured presentation on a relevant topic to each phase of implementation plus unstructured time for sites to ask questions and give feedback to each other. The Teams channel will provide an outlet to communicate asynchronously with individuals from different cohorts.
High-touch/enhanced REP (resources available to non-adopting and/or non-sustaining sites)	Foundational resources	No change to accessing resources outlined above as part of low-touch support.
	Technical assistance	Direct access to Function QUERI implementation facilitators for technical assistance as needed.
	External facilitation	Tailored, one-on-one calls approximately every 3 to 4 weeks between site implementation teams and a trained practice facilitator with the goal of promoting interactive problem solving in the context of a supportive interpersonal relationship. Discussion may focus on key barriers to implementation, available assets to leverage, and actionable tasks to monitor and improve delivery.

### Foundational REP implementation support (low-touch)

For this application, our low-touch approach has been conceptualized as a bundle of implementation strategies because it involves integrated yet discrete resources selected to address identified barriers to implementation success [40]. Foundational support features standardized self-guided materials, resources, and a toolkit to support implementation and site-specific adaptations. Additional details on the process of developing these materials are described in Table 2 and elsewhere [41].

### Enhanced REP implementation support (high-touch)

Although an approach like REP is efficient and evidence based, it is not designed to address differences in organizational readiness or capacity to implement change, which may inhibit EBP adoption. Function QUERI addresses this potential limitation by supplementing foundational support with enhanced implementation support that is tailored to sites who do not meet Group PT program benchmarks and may benefit from extended support.

High-touch strategies feature the use of practice facilitation, defined as a multicomponent approach to improve the capacity of sites to address care quality and implementation gaps [42]. High-touch facilitation includes interactions with a trained implementation specialist (IS). Training emphasizes best practice of evidence-based implementation facilitation techniques, such as building relationships with and between others, creating an infrastructure for support and problem solving, and encouraging processes to monitor program progress [43]. Despite heterogeneity of this role in the literature, it has shown to be effective in supporting implementation for a range of programs and behavioral health interventions [44–46].

Sites that receive enhanced support will be engaged in one-on-one calls approximately every 3 to 4 weeks with an IS from the study team. The IS will coach individual sites using techniques, processes, and activities to help teams make decisions and identify and solve problems. Facilitators’ recommendations will be tailored to individual site’s needs and context. For example, if a site wishes to increase the number of consults being received then the facilitator will gather information about past or ongoing efforts and then suggest additional marketing and education activities.

## Group PT site recruitment and onboarding

### Site inclusion criteria, recruitment, and onboarding

Sites must meet the following criteria to enroll in the study: (1) clinical personnel on staff to conduct initial evaluations and lead group classes (e.g., physical therapist, PT assistant, or kinesiotherapist): this includes at least 1 primary clinician and 1 back-up clinician to cover all aspects of program delivery, (2) Offer outpatient PT service, and (3) Space to conduct group sessions (if implementing in-person Group PT classes). Sites currently offering a group class specifically for knee OA will be ineligible. Sites will be asked to commit to 12 months of Function QUERI activities, use the pre-programmed electronic health record (EHR) templates, and provide the staff necessary to implement Group PT. Site recruitment will include presentations to national and regional VA network calls and information sent through VA rehabilitation listservs. We will also identify and reach out to rural VA sites with a high volume of PT referrals to Community Care, which may signal a need for an efficient care model for delivering PT services.

We will conduct individual, virtual meetings with sites expressing interest in the program to provide an overview of program benefits, expectations, and resources provided by the Function QUERI team. Sites will be required to return participation agreements, signed by the site PM&RS Chief and a designated local point of contact (POC) for the program. Following receipt of the participation agreement, sites will provide information, via an intake form, on all personnel intending to participate in Group PT program delivery and implementation (e.g., clinicians, schedulers, supporting leadership). Sites will be onboarded in cohorts ranging in size from 5 to 7 sites with an assigned launch date. The cohort size range was selected because it is appropriate for delivering components of low-touch implementation support. On launch day, each site will receive access to the toolkit and all team members will be added to a Microsoft Teams chat group for collaboration and networking. A welcome call will be conducted shortly after the launch date to orient sites to the available resources and timeline for future activities. The Function QUERI study team will also transfer the pre-developed EHR templates for collecting patient outcomes.

## Data collection and measures

All measures, descriptions, and data collection time points are detailed in Table 3.

**Table 3:** Study outcomes

Outcome	Description	Data source	Assessment timepoint(s)
*Implementation outcomes (site level)*			
Penetration (primary)	Average number of patients enrolling in the EBP monthly	Administrative and VA electronic health record (VA Corporate Data Warehouse)	6 months, 12 months (primary)
Adoption (dichotomous)	Delivery of Group PT as a clinical service (at least 1 class) and enrollment of at least 5 patients		
Fidelity	Average number of sessions attended by patients who enroll in the EBP		
*Effectiveness outcomes (patient level)*			
30-s chair rise	Number of times patient can stand up from a chair and sit back down within a 30-s period	Collected by clinicians at time of patient visit via electronic or paper survey and stored in the VA electronic health record (VA Corporate Data Warehouse)	Every Group PT class *Note: patient satisfaction and ability to deal with daily problems regarding knee pain are asked after the first class*
Pain experienced during chair rise	Patient-reported pain experienced during chair rise (0–10 numeric scale)		
Patient-reported physical function	PROMIS Short form 4a		
Patient-reported pain interference	PROMIS Short form 4a		
Patient satisfaction	Patient’s response to: “How satisfied are you with the Group PT program?” on a numeric rating scale (0–10)		
Ability to deal with knee pain	Patient’s response to: “Compared to before you before you started the Group PT program, how would you rate your ability to deal with daily problems with knee function and pain now?” on a 5-point Likert (from much worse to much better)		
*Implementation context and experience*			
Program adaptations (using Wiltsey Stirman’s FRAME framework) [47]	Sites report changes to program staffing, delivery, equipment, education, and documentation	Site self-report	Baseline, 6 months, and 12 months
Qualitative interviews	Implementation process and experience	Key informant semi-structured interviews	Baseline (all sites), 6 months (all sites), and 12 months (sites that receive enhanced support)
Organizational Readiness for Implementing Change (ORIC) [48]	Used to determine organizational members’ change commitment and efficacy. Each item includes a Likert scale from 1 (disagree) to 5 (agree).	Staff-reported	Baseline
Organizational resilience^a^ [49]	Measure of the capacity of an organization to anticipate, prepare for, respond, and adapt to both incremental changes and sudden disruptions to survive and thrive.		Baseline and 12 months
Challenging and helpful implementation factors	Using a list of items developed through previous work by the Function QUERI team, respondents are asked to select how challenging or helpful resources and other factors (e.g., patient awareness) will be or were when implementing Group PT at their facility on a scale of 1 (not at all) to 5 (very).		Baseline and 12 months
Implementation Climate Scale (ICS) [50]	Captures six dimensions of the organizational context that indicate to employees the extent to which their organization prioritizes and values the successful implementation of Group PT.		Baseline
Implementation process and experience	Questions regarding the use of the different Group PT implementation resources and activities. Respondents are asked to indicate, whether their facility used the tool, how much, and how helpful it was for supporting implementation of Group PT.		12 months
Program Sustainability Index^b^ [51]	A measure of a program’s capacity for sustainment over time. Using a list of statements related to common program attributes (e.g., leadership competence and staff involvement and integration), respondents are asked to what extent the statements are reflective of their facility on a scale of 0 (not at all) to 4 (to a very great extent).		12 months
COVID-19 impact question	What impact do you think the COVID-19 pandemic and its effects had on your facility's ability to implement the Group PT program?		Baseline and 12 months
Enhanced support experience^c^	How helpful were the enhanced support sessions with Function QUERI staff? From 1 (not at all helpful) to 5 (very helpful).		12 months
	What, if anything, would you change about the enhanced support sessions? [free text response]		
Demographic measures	Questions related to job title, years at the VA, age, gender, etc		Baseline and 12 months
*Cost*			
Clinical delivery team costs	How much time did each delivery team member spend conducting the following tasks for each Group PT class? • Time entering data in the EHR • Time spent with patients on clinical issues outside of class • Time spent outside of class assisting patients with technical issues • Other time associated with preparing for delivery of Group PT	Site self-report	After sites host their first Group PT class
Implementation strategy costs	During the month before Group PT launched, on an average week how much time did each team member spend planning for and/or supporting your Group PT program?	Site self-report	

^a^Subset of measures–items on adaptive capacity rather than the full 13-item short form

^b^Slight wording change to Q2 and rewording of Likert scale categories (per author’s recommendation)

^c^Sites that receive enhanced support will also be asked about their experience. Except for these questions, all staff, regardless of study arm, receive the same pre-and post-implementation survey questions

### Implementation outcomes

Implementation outcomes were selected using the taxonomy defined by Proctor and colleagues [37] and based on the overall goals of Function QUERI and operational partners. They include *penetration*, defined as the level of integration of a practice within a service setting, *adoption*, or the initial actions to employ a novel practice, and *fidelity*, the degree to which the program is implemented as it was designed or intended. These metrics will be assessed from the VA’s EHR [52], including Group PT-specific EHR templates.

### Implementation context and experience

#### Semi-structured interviews

We will conduct 30-min individual or group semi-structured virtual interviews with key informants, identified on the intake form or by other team members through snowball sampling, to elicit a description of the facilitators and barriers that affected their implementation of Group PT. Specifically, we will ask about detailed activities used and any additional strategies developed during program implementation, probing for details based on Proctor’s criteria for specifying and reporting strategies (e.g., actor, action, target, temporality, frequency). We will sample 50% of sites per cohort for interviews to maximize site diversity based on implementation experience (5-point Likert from none to quite a lot) and rurality (serving ≥ 50% or < 50% rural/highly rural Veterans). We will aim to conduct 5–10 interviews per sampled site at baseline and 6 months. Only sites that receive enhanced support will be asked to participate in interviews at 12 months. All interviews will be recorded with the permission of the interviewee.

#### Quantitative surveys

To capture salient contextual factors related to Group PT implementation, surveys will be administered at baseline (pre-implementation) and at 12 months (post-implementation). Surveys will include validated measures (outlined in Table 3) to capture implementation process and factors influencing implementation as outlined in our overarching framework (e.g., characteristics of the intervention, site’s environmental context, and team functioning). All staff members listed on the intake form will be invited using VA REDCap (Research Electronic Data Capture) to complete surveys [53]. If additional program support staff are hired or identified later by the local POC then they will also be included. Each staff member will receive a survey invitation plus two reminders (3 contacts). To increase survey participation, we will offer a raffle for small prizes (< $50). Additionally, Group PT program adaptations will be reported at baseline, 6 months, and 12 months using Wiltsey Stirman’s FRAME [47], which provides a standardized way to track modifications and facilitate continual monitoring. Only the local POC will receive the adaptations form, but we will encourage teams to complete the form together. Each POC will receive a survey invitation plus two reminders (3 contacts).

### Effectiveness outcomes

Effectiveness measures were selected with consideration of outcomes that are of high importance to patients with knee OA and their health care providers, as well as feasibility of administration and documentation. These measures are collected at each Group PT class and include both self-report items and physical performance tests, as shown in Table 3.

### Cost data

We are also collecting two types of cost data: clinical delivery and implementation strategy costs. Within 2 weeks after a site has started offering Group PT as a clinical service, the local POC will be emailed two Excel forms to document this data. The implementation strategy form will measure time spent training and preparing for Group PT delivery during the month prior to sites offering their first Group PT class. The clinical delivery form, used to measure personnel time associated with actual Group PT delivery, will involve tracking time associated with several tasks during the first 6 weeks of program delivery. Both implementation and delivery costs will be associated with the individual who is completing each task (e.g., PTA, PT) and their salary to quantify time in monetary terms. Lastly, the cost of any purchased equipment will be documented by the local POC.

## Analysis

### Quantitative analysis

As part of our hybrid type III effectiveness-implementation study design, the primary research question compares differences in implementation outcomes between arms. Implementation outcomes are continuous (penetration, fidelity) and binary (adoption) cluster-level outcomes, and generalized linear models will be used to examine the effect of REP versus the addition of enREP on implementation outcomes at 12 months [54]. The main predictor of interest will be REP versus enREP, with indicators for the stratification variables of complexity level and rurality included in the final model. In secondary analyses, implementation outcomes at 6 months will be assessed. We will examine how implementation outcomes change over time using descriptive methods (e.g., plots, descriptive statistics, subgroups). We will describe patient-level effectiveness outcomes overall and by study arm. We will examine how patient outcomes change over time using descriptive methods (e.g., plots) and will calculate descriptive statistics for all visits and change outcomes among patients who have data from multiple visits.

Descriptive statistics for staff survey measures related to implementation context and experience (Table 3) will be calculated overall and by study arm. We will use the same modeling approach described above to examine the effect of implementation package on survey measures. In addition, we will assess the relationship of implementation context and experience measures on implementation and effectiveness outcomes.

### Qualitative analysis

We will use directed content analysis [55] that includes (1) a priori codes to indicate foundational, low-touch activities and ERIC (Expert Recommendations for Implementing Change) implementation strategies [56, 57] (e.g., “engagement with toolkit”) and or (2) data derived codes of barriers to implementation.

### Integration of qualitative and quantitative data

We will summarize the coded data in a framework matrix [58] to compare reports of implementation strategies and barriers across sites and by study arm and implementation outcomes. The rows of the matrix will reflect coded implementation strategies and the columns will reflect the study arm and implementation outcomes for each site. Summaries of coded data within each matrix cell will describe the implementation strategy or barrier. The qualitative researchers will meet with the project director and other team members to review the matrix and identify patterns between sites with lower versus higher quantitatively assessed implementation outcomes.

### Sample size and power

Sample size calculations were conducted for the primary implementation outcome penetration at 12 months. Using a two-sided *t* test based on a sample size of 16 sites (randomized 1:1 to each study arm), a type-1 error rate of 5%, we will have 80% power to detect an effect size difference of 1.5 and 90% power to detect an effect size difference of 1.7 between arms. Based on data of mean number of patients enrolling per month over a 1-year period of implementation of Group PT in Durham, we assumed standard deviations ranging from 1.5 to 2.5. Therefore, the effect size differences were powered to detect differences in mean number of patients enrolling per month between arms of 2.3 to 3.8 patients for 80% power and 2.6 to 4.3 patients for 90% power.

### Business Case Analysis (BCA)

The base-case BCA will compare the expected value for costs (implementation and delivery), implementation outcomes, and effectiveness outcomes between treatment arms using site level estimates from the trial data. We will model the probabilities meeting benchmarks and associated outcomes (costs, penetration) occurring with those events using a decision tree. In the low-touch arm, sites will not receive enhanced support when benchmarks are not met, though costs of foundational support will be incurred and tracked in the model. In the high-touch arm, sites will receive enhanced support when benchmarks are not met and will incur additional implementation costs associated with enhanced support. This will allow us to compare the expected costs and penetration in the cohort of sites if all sites had received high-touch support versus not. In addition, we will use one-way and probabilistic sensitivity analysis to simulate likely outcomes in the context of distributions informed by the trial data as well as prior evidence for the EBP. By modeling plausible scenarios, the decision model will allow us to communicate a practical range of estimates to sites and operational partners rather than relying on statistical significance. Monte Carlo probabilistic sensitivity analysis will be used to incorporate measured uncertainty in the estimates for all outcomes and generate probabilities for exceeding VA thresholds for value. We will work with our operational partners to establish thresholds for decision-making [59].

## Discussion

Although there have been many RCTs of PT and exercise-based interventions for knee OA, there have been very few implementation studies in this area and none, to our knowledge, comparing different approaches to implementation support. As a result, our knowledge regarding optimal implementation activities for scaling PT interventions and care models in real-world clinical settings is limited. Our team has worked closely with clinical and operational partners on a multi-step journey to study and implement Group PT. Our initial RCT incorporated pragmatic elements that facilitated the transition to more implementation-focused work [26]. In particular physical therapists at the DVAHCS delivered the Group PT program, and it was embedded into their clinical workflow. This provided an opportunity to assess feasibility of integration into the PT Service, and physical therapists provided direct input on the practical and clinical aspects of the program. When the DVAHCS PT Service began implementing Group PT following completion of the RCT, we were able to conduct a robust evaluation of the program [28]. This evaluation led to EBP refinements and provided our team with experience in collection of both patient-reported and EHR-based outcomes in the context of Group PT delivery. These experiences with the initial Group PT RCT and the DVAHCS evaluation have led to a rigorous, partner-informed implementation trial.

There are advantages to a group-based PT model for both health systems and patients. For health systems, the efficiency and potential cost savings of Group PT are clear advantages. For a six-session round of Group PT with 10 enrolled Veterans, a PT service would provide 60 patient-hours of care with 6 h of clinician time. This represents substantial time and resource savings. For patients, use of a group-based model can improve access, particularly in health care settings that have limited PT personnel resources. Another advantage of the Group PT program is the standardized approach to delivering exercise-based PT for knee OA. We have developed this program based on research and best practices for PT and exercise for knee OA, and sites implementing Group PT have access to many ready-to-go resources for program delivery.

There are also some challenges to implementing group-based programs. First, starting a new group program requires logistical tasks such as setting up new clinics or note templates in the EHR and establishing scheduling procedures. Second, the efficiency of group programs depends on continued enrollment of patients to fill the classes. Sites therefore need plans for maintaining referrals of patients to the program. Similarly, sites need plans to minimize “no shows” to maximize program efficiency and impacts. Third, although all patients enrolled in Group PT have knee OA, they vary in terms of physical function, comorbidities, and other factors that require tailoring of the exercise approach. Although this can be a challenge to delivering PT in a group setting, we have designed Group PT with this heterogeneity in mind and provided sets of exercises appropriate for patients with different functional abilities.

This study will also yield scientific advances related to strategic tailoring of support for implementing new EBPs. Some prior studies have evaluated adaptive implementation approaches, in which sites are provided with more intensive support if they do not meet benchmarks [60–62]. However, research in this area is still limited. Our Function QUERI projects will provide robust evidence regarding the use of implementation intensification across three different EBPs. To our knowledge, the Group PT RCT is the first to examine a tailored implementation approach for delivering a PT-based intervention. We are collecting detailed data on adaptations to Group PT, which will enhance understanding of how sites deliver the program in a way that ensures best fit with local resources, needs, and structures. We are also collecting robust information on site-level barriers and facilitators to implementation. These data will enhance our understanding of the specific preconditions, site-level adaptations, and tailoring of support that contributes toward more widespread implementation of Group PT. Additionally, the planned analysis on the costs of each implementation strategy, while also assessing the impacts on implementation outcomes, presents a unique opportunity to provide guidance to leadership and policy makers on how to cost-effectively promote adoption and sustainment of Group PT. While implementation researchers have made notable progress in the design and testing of strategies to improve implementation, comparative economic evaluation of implementation strategies are lacking. Yet, these findings are critical for payers, policy-makers, and providers to make informed decisions on whether specific strategies are an efficient use of resources. Currently, few implementation studies include implementation cost data and even fewer compare strategies’ cost effectiveness [63].

There are some limitations to this trial. First, sites are followed for 12 months after entry into the study, and it will be important for future work to evaluate sustainment of Group PT delivery over a longer period. Second, patient outcomes are collected through the end of Group PT participation, and future work should also examine patient-level effectiveness and maintenance at later time points. Third, although we aim to recruit sites that vary in terms of geography, rurality, and facility complexity, our final sites may not be representative in all ways. In particular, participating sites may have a higher level of organizational readiness to change or other motivators that make implementation success more likely. Fourth, our study only includes VA sites, and additional work is needed to examine Group PT effectiveness and implementation in other health systems.

In summary, our Function QUERI program is poised to have significant impacts for Veterans at high risk for negative health outcomes, health systems seeking to efficiently implement EBPs and the implementation science community. The Group PT trial is addressing one of the most common, function-limiting health conditions among Veterans and older adults in general. Based on our prior work [26, 28], the Group PT program has strong potential to become a standard offering for PT Services and clinics, improving function and pain-related outcomes for patients with knee OA. This trial will take important steps toward that goal.

### Supplementary Information

**Additional file 1.**

## Data Availability

Not applicable.

## Abbreviations

OA: Osteoarthritis
VA: Veterans Affairs
PT: Physical therapy
RCT: Randomized clinical trail
VAMCs: Veterans Affairs Medical Centers
REP: Replicating Effective Programs
EnREP: Enhanced REP
DVAHCS: Durham VA Healthcare System
Function QUERI: Optimizing Function and Independence Quality Enhancement Research Initiative
EBP: Evidence-based clinical program
EHR: Electronic Health Record
PM&RS: Physical Medicine and Rehabilitation Service
IS: Implementation specialist
POC: Point of contact
Group PT: Group physical therapy for Veterans with knee osteoarthritis
BCA: Business case analysis
